# Renal ultrasonographic shear-wave elastography and urinary procollagen type III amino-terminal propeptide in chronic kidney disease dogs

**DOI:** 10.14202/vetworld.2020.1955-1965

**Published:** 2020-09-23

**Authors:** Chutimon Thanaboonnipat, Saikaew Sutayatram, Chollada Buranakarl, Nan Choisunirachon

**Affiliations:** 1 Department of Veterinary Surgery, Faculty of Veterinary Science, Chulalongkorn University, Bangkok, 10330, Thailand; 2 Department of Veterinary Physiology, Faculty of Veterinary Science, Chulalongkorn University, Bangkok, 10330, Thailand

**Keywords:** chronic kidney disease, dog, renal fibrosis, shear-wave elastography, urinary procollagen type III amino-terminal propeptide

## Abstract

**Background and Aim:**

Renal fibrosis is a well-established pathological alteration associated with chronic kidney disease (CKD) in several species and progresses as CKD advances. Although a renal biopsy is the gold standard for determining renal fibrosis, it is an invasive, impractical method for clinical practice. In humans, ultrasonographic shear-wave elastography (SWE), a novel advanced diagnostic imaging tool, can evaluate renal parenchyma stiffness, and urinary procollagen type III amino-terminal propeptide (uPIIINP), a promising renal fibrosis biomarker in humans, has increasingly been use applied to reduce the biopsies. This study compares renal tissue elasticity observed through SWE Young’s modulus (E) values between healthy dogs (HD) and those with CKD.

**Materials and Methods:**

The E value acquired by SWE, uPIIINP levels, and renal function were evaluated in 15 CKD dogs and 15 HD.

**Results:**

The renal cortical E values were significantly higher than the renal medullary E values in both groups (p<0.001). Renal cortical and medullary E values in CKD dogs were significantly higher than in HD (p<0.01). Cortical E values had greater significant correlations with renal functional parameters than the medullary E values and had a significant positive correlation with concentrations of plasma creatinine (Cr) (p<0.001); blood urea nitrogen (p<0.01); urine protein Cr ratio (p<0.01); and fractional excretions of sodium (p<0.05), potassium (p<0.05), chloride (p<0.05), and magnesium (p<0.001) while they had a negative correlation with urine specific gravity (p<0.05) and urine osmolality to plasma osmolality ratio (p<0.05). The uPIIINP to Cr (uPIIINP/Cr) ratios of CKD dogs were higher than those of HD (p<0.001). Moreover, the uPIIINP/Cr levels presented significant correlations with the renal cortical E values (p<0.01) and also the renal functional parameters.

**Conclusion:**

SWE offers a complementary, non-invasive diagnostic imaging tool for evaluating renal tissue stiffness in CKD dogs with renal function deterioration. In addition, uPIIINP levels are associated with renal function and structural changes in dogs. Therefore, the uPIIINP level might be a non-invasive, complementary, and promising biomarker for evaluating renal fibrosis in canine CKD.

## Introduction

Chronic kidney disease (CKD), the most common renal disorder in dogs, is an irreversible and progressive impairment of kidney structure or function from permanent injury that has exceeded the maximal capacity of compensation, lasting for at least 3 months [1-4]. CKD encompasses a wide variety of kidney problems either congenital or acquired problems, of which the initiating cause of the CKD usually cannot be identified [1,3]. The commonly reported causes of canine CKD include incomplete recovery from an acute renal injury, pyelonephritis, glomerulonephritis, tubulointerstitial disease, neoplasia, hypercalcemia, and various hereditary nephropathies [1-5]. The prevalence of canine CKD in the United Kingdom was reported to be approximately 0.5-1.5% [5]. Demographic predisposing factors of canine CKD consist of advancing age [6-8], male sex [2], and breed including Chinese Shar-Pei [9], Bull Terrier [10], English Cocker Spaniel [11], West Highland White Terrier [12], Boxer [13], and mixed breeds [2]. Renal fibrosis, especially tubulointerstitial fibrosis, is an irreversible and progressive pathological change reported to occur with CKD in both humans and dogs [14-17] and is a main pathological change during the CKD advancement [16,17], subsequently leading to impairment of renal functions. Therefore, early diagnosis facilitating early treatment is important to slow CKD progression [18-20]. Early clinical signs of canine CKD are usually non-specific [3]. Although measuring the glomerular filtration rate (GFR) is considered the gold standard for evaluating renal function, methods for GFR assessment are technically cumbersome and not practical for clinical implementation [21]. The blood creatinine (Cr) concentration is commonly used for screening impaired renal function. However, Cr is a non-specific marker, because it can be affected by various factors, including sex and muscle mass [21-23]. B-mode ultrasonography is a common diagnostic imaging modality that can be used to evaluate anatomical changes in the kidneys, including size, shape, and contour. Notably, the normal ratio of renal length to the aortic luminal diameter (K/Ao) in normal dogs ranges from 5.5 to 9.1 [24], while this ratio is lower in dogs with advanced CKD [25].

Many renal fibrosis biomarkers are currently used to evaluate renal fibrosis in humans, but there is still no precise method that can replace a renal biopsy [26,27]. However, a renal biopsy is an invasive procedure with several complications; therefore, it is not performed in routine clinical practice, particularly in high-risk patients such as dogs with CKD [28]. Urinary procollagen type III amino-terminal propeptide (uPIIINP) has been developed as a promising renal fibrosis biomarker in humans [16,29]. The uPIIINP has been reported to be one of promising biomarkers in humans that can indicate the degree of renal fibrosis and CKD progression [16,30,31]. In veterinary medicine, it has recently been reported that the uPIIINP to Cr (uPIIINP/Cr) ratio was significantly higher in cats with CKD than in healthy cats [32]. Furthermore, the plasma PIIINP levels have been utilized as a fibrosis marker for assessing cardiac remodeling [33], idiopathic pulmonary fibrosis [34], and liver fibrosis in dogs [35]. In addition, the advanced diagnostic imaging techniques, particularly renal ultrasonographic shear-wave elastography (SWE), have been increasingly performed in humans [36,37]. Increases of both cortical and medullary renal echogenicity detected by B-mode ultrasound are related to the CKD lesions [38]; these increases may also be present in dogs without evidence of renal impairment [39]. SWE can evaluate renal parenchyma elasticity or renal tissue stiffness [40,41] by facilitating the Young’s modulus (E) value measurements [42]. The previous human and feline studies have shown that renal E values of humans and cat CKD patients are significantly higher than those of healthy persons and cats [32,37]; moreover, the renal E values also correlate with decreases in renal function in CKD patients [32,36].

In veterinary medicine, the information about the uPIIINP biomarker in CKD dogs has not been previously reported and the SWE information is currently only available in dogs with normal kidneys [43].

Therefore, the aims of this study were, first, to compare the renal tissue elasticity observed through E values, acquired by SWE, between healthy dogs (HD) and CKD dogs; second, this study evaluated the relationships between the renal tissue stiffness and functional renal parameters, including the plasma Cr concentration, blood urea nitrogen (BUN), urine specific gravity (USG), the urine protein Cr (UPC) ratio, and the urine osmolality to plasma osmolality (Uosm/Posm) ratio as well as, the fractional excretion of sodium, potassium, chloride, and magnesium (FE_Na_, FE_K_, FE_Cl_, and FE_Mg_) in both groups. Third, this study compared uPIIINP levels between HD and CKD dogs and determined the relationship between uPIIINP/Cr ratios and renal E values.

## Materials and Methods

### Ethical approval and informed consent

This study was approved by the Chulalongkorn University Animal Care and Use Committee, Faculty of Veterinary Science, Chulalongkorn University (Protocol number: 1731055) and all participating dog owners signed consent forms. This study was performed within a visit day and then all client-owed dogs were allowed to go back home.

### Study location and period

This study was performed using client-owned dogs that presented to the Small Animal Teaching Hospital, Faculty of Veterinary Science, Chulalongkorn University between September and December 2017.

### Animals and experimental design

The dogs which age-matched were assigned to either the HD (HD; n=15) or CKD dogs (CKD; n=15) groups. Dogs with normal findings based on history, physical examination results, and routine blood examinations, including complete blood counts (CBC) and plasma biochemistry, USG and UPC ratio as well as abdominal radiography, and B-mode abdominal ultrasound were considered to be HD. CKD dogs were defined based on the International Renal Interest Society (IRIS) staging system that defined CKD based on a blood Cr level of more than 1.4 mg/dl for more than 3 months; these levels were considered stable on being considered for inclusion [44]. All CKD dogs were subjected to the same diagnostic procedures as the HD group. Dogs presenting any signs of ascites, infectious diseases, lymphoma, or congenital kidney diseases, such as polycystic kidney, nephrolith, and hydronephrosis were excluded from this study.

On the day of the visit, the blood samples were collected from all dogs in tubes containing either of ethylene diamine tetra-acetic acid (EDTA) (K3 EDTA collection test tube, FL MEDICAL, Italy) for CBC measurement or lithium heparin (VACUETTE^®^, Greiner Bio-One GMbH, Germany) for determining the concentrations of Cr, BUN, alanine aminotransferase (ALT), alkaline phosphatase (ALP), total protein, and albumin. Some of the collected plasma was centrifuged and stored at −20°C for analysis of plasma osmolality and electrolytes (Na^+^, K^+^, Cl^-^, and Mg^2+^). Microscopic observation of urine sediment was performed before measuring urine protein levels, to exclude canine patients with urinary tract infections. In addition, urine samples were collected and stored at −20°C for evaluations of the concentrations of Cr, protein, PIIINP, and electrolytes (Na^+^, K^+^, Cl^-^, and Mg^2+^) concentrations as well as osmolality. Indirect systolic blood pressure was measured in triplicate at the proximal hind limb or distal forelimb in each dog, and the average value was used.

### Analytical procedures

Plasma and urine concentrations of Cr, BUN, ALT, ALP, total protein, and albumin were analyzed by an automated analyzer (The IL ILab 650 Chemistry Analyzer, Diamond diagnostic, USA). Frozen plasma and frozen urine samples (−20°C) were thawed to measure the osmolality and electrolytes concentrations. Posm and Uosm were measured by an automatic cryoscopic osmometer (OSMOMAT^®^ 030, Gonotec GmbH, Germany). Plasma and urine electrolyte concentrations were determined by an automated machine (ARCHITECT c16000 clinical chemistry analyzer, Abbott Laboratories Ltd, USA) and an automated analyzer (COBAS INTEGRA^®^ 400 plus, Roche Diagnostics Ltd., Switzerland), respectively. USG was determined using a refractometer (Master Refractometer, ATAGO^®^, Japan). The urine protein level was determined after protein precipitation with 3% sulfosalicylic acid [37]. The FE_Na_, FE_K_, FE_Cl_, and FE_Mg_ were calculated as previously described [45]. Systolic indirect blood pressure was measured using a Doppler blood pressure instrument (Vet-Dop 2™, Vmed Technology, USA). The concentrations of uPIIINP were determined in frozen urine samples (−80°C) in duplicate using a double sandwich enzyme-linked immunosorbent assay (ELISA) kit (Canine Procollagen Type III N-Terminal Propeptide (PIIINP) ELISA Kit, Cat. No. MBS2608207; MyBioSource, USA), following the manufacturer’s instructions. The intra-essay and inter-essay coefficient of variation were <12%. The uPIIINP/Cr ratios were calculated and expressed as ng/mgCr.

### B-mode ultrasonography and SWE procedure

**Figure-1:**
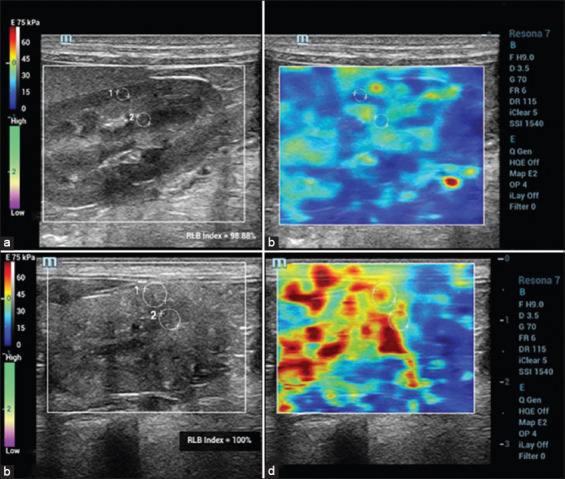
B-mode ultrasound images (a and c) and color quantitative elastogram superimposed on the B-mode with regions of interest (b and d) between a healthy dog (A and B) and a chronic kidney disease dog (c and d).

The ultrasonographic examinations were performed without any sedation. Dogs were manually restrained and positioned in either right or left lateral recumbency for each ipsilateral renal observation. Hair was clipped and acoustic gel was applied to the skin in preparation for the ultrasound procedure. B-mode ultrasonographic images were obtained using a 9 MHz-bandwidth of linear transducer (Resona-7, Mindray Medical International, China) to screen the overall abdominal organs including the kidney. The K/Ao ratio was assessed by measuring the maximal length of each kidney, in the sagittal plane, as well as the aortic luminal diameter, as previously described [24]. All measurements were performed twice, and the results were then averaged for analysis. Then, SWE was examined by the same instrument on the sagittal plane of the kidney. This instrument uses the Sound Touch Elastography technology that an elastogram displays as a dual-screen mode of a B-mode image and a color quantitative elastogram. The elastogram expresses a red color to represent the hardest tissues whereas a blue color represents the softest tissues. During the SWE procedure, a transducer is carefully placed over the kidney with a small amount of pressure to maintain high reliability (RLB). The RLB map indicates the SWE image quality, presented as percentages, and elasticity results from the high RLB of shear-wave images are more reliable. The fixed region of interest was adjusted as wide as possible to cover both renal cortex and renal medulla. The Young’s modulus (E) value in kilopascal unit was selected to represent renal parenchyma elasticity; a high E value indicates stiff parenchyma, while lower E value signifies soft parenchyma [42,46]. Three separated the elastograms were selected and renal E values were measured at the nearest to a probe and central renal parenchyma using a circular electrical caliper with the same size at the renal cortex and medulla in both of HD and CKD dogs (Figure-1); finally, then renal E values were averaged.

### Statistical analysis

All data analyses were performed using GraphPad Prism 7^®^ software (GraphPad Software, CA, USA). The results are expressed as mean±standard deviations. The normality of the data distribution was tested with the Shapiro–Wilk test. The average E values between the left and right kidneys in each group were analyzed by paired t-test. The average E values and uPIIINP/Cr ratios were compared between groups using the unpaired t-test or Mann–Whitney test. Pearson’s correlation coefficient and Spearman’s correlation coefficient were used to investigate the correlations between parameters. Statistical significance was considered if p<0.05.

## Results

The geographic data of HD and CKD groups are presented in Table-1. The mean ages of both groups in this study were age-matched. The HD group consisted of Shih Tzu (7), Yorkshire Terrier (3), Siberian Husky (1), Cardigan Welsh Corgi (1), Cocker Spaniel (1), Poodle (1), and Chihuahua (1), while the CKD group encompassed Shih Tzu (5), Chihuahua (3), Mixed breed (3), Siberian Husky (1), American Pitbull Terrier (1), and Domestic Thai (2). The CKD dogs were classified as either IRIS Stage 2 (eight of 15 dogs, 53.3%) or Stage 3 (seven of 15 dogs, 46.7%).

**Table-1:** Demographic information of HD and CKD dogs.

Parameters	HD group (n=15)	CKD group (n=15)
Sex		
Female (spayed)	10 (1)	4 (2)
Male (castrated)	5 (1)	11 (3)
Age (years)	9.2±3.6 (2-15)	11±5.4 (2-21)
Body weight (kg)	9.8±9.3 (1.7-35.0)	11.5±11.7 (1.2-39.4)
Body condition score	3.3±0.5 (2.5-4.0)	3.0±0.6 (2.0-4.0)

Data are presented as mean±SD and range. HD=Healthy dogs, CKD=Chronic kidney disease

B-mode ultrasonographic findings showed that the kidneys of dogs in the HD group had a normal shape, contour, echogenicity, and echotexture, with a mean renal length, for both right and left kidneys, of 4.0±1.0 cm. The mean K/Ao ratios of the HD group were 7.3±1.2 and 7.3±1.1 for the right kidney length to aorta diameter (RK/Ao) and the left kidney length to aorta diameter (LK/Ao), respectively. In contrast, the kidneys of the dogs in the CKD group demonstrated increased cortex and/or medulla echogenicity, poor corticomedullary demarcation, and an irregular contour, with a mean renal length of 3.4±1.6 and 4.0±1.3 cm for the right and the left kidneys, respectively. The mean K/Ao ratios of CKD dogs were 5.7±2.0 and 6.5±1.3 for the RK/Ao and LK/Ao, respectively. The average renal lengths were not significantly different between groups. However, the average K/Ao ratios of CKD dogs were significantly lower than those of HD (RK/Ao; p=0.005 and LK/Ao; p=0.036).

The average blood pressure of the HD group was significantly lower than that of the CKD group (123.5±22.6 mmHg and 141.0±22.1 mmHg, respectively, p=0.045). The renal functional parameters, including the plasma concentration of Cr, BUN, USG, the UPC ratio, the Uosm/Posm ratio, and the FE_Na_, FE_K_, FE_Cl_, and FE_Mg_ were compared between the groups and are presented in Table-2. All renal functional parameters in the CKD group were significantly different from those in the HD group. Plasma concentrations of Cr, BUN, and the UPC ratio of CKD dogs were significantly higher than those in HD (p<0.001), while USG and the Uosm/Posm ratio of CKD dogs were significantly lower than those of HDs (p<0.001). The FE of electrolytes (FE_e_) were significantly higher in CKD dogs than in HD (FE_Na_, FE_K_, FE_Cl_, and FE_Mg_: p<0.001, and FE_K_: p=0.001).

**Table-2:** Comparison of the renal functional parameters including plasma Cr concentrations, BUN, USG, UPC ratio, Uosm/Posm ratio, FE_Na_, FE_K_, FE_Cl_ and FE_Mg_, and the anatomical renal length parameter including RK/Ao and LK/Ao between the healthy dogs (HD) and chronic kidney disease dogs (CKD) and correlations between the average uPIIINP/cr ratios and those parameters.

Parameters	HD group	CKD group	Reference intervals	Correlations with average uPIIINP/cr ratios

				r
Renal function				
Plasma Cr (mg/dl)	0.9±0.2***	2.4±1.0***	0.3-1.4***	0.757***
BUN (mg/dl)	20.0±6.5	44.8±20.7***	5-21***	0.662***
FE_Na_ (%)	0.2±0.2***	2.7±2.1***	<1***	0.858***
FE_K_ (%)	13.3±7.1***	42.5±31.8**	<20***	0.688***
FE_Cl_ (%)	0.5±0.2***	4.2±2.6***	<1***	0.862***
FE_Mg_ (%)	2.4±2.3***	18.4±16.7***	<5.4	0.754***
USG***	1.045±0.01***	1.022±0.007***	1.035-1.060	−0.776***
Uosm/Posm ratio	4.0±1.2*	1.3±0.8***	NA***	−0.829***
UPC ratio**	0.04±0.07**	2.20±3.20***	<0.2^a^	0.128
Anatomical renal length				
RK/Ao	7.3±1.2	5.7±2.0**	5.5-9.1**	0.473*
LK/Ao	7.3±1.1***	6.5±1.3*	5.5-9.1****	0.411*

a Reference of UPC ratio in dogs=Non-proteinuric (<0.2), borderline (0.2-0.5), proteinuric (>0.5). BUN=Blood urea nitrogen, Cr=Creatinine, FE_Na_=Fractional excretion of sodium, FE_K_=Fractional excretion of potassium, FE_Cl_=Fractional excretion of chloride, FE_Mg_=Fractional excretion of magnesium, USG=Urine specific gravity, Uosm/Posm ratio=Urine osmolality per plasma osmolality ratio, UPC ratio=Urine protein creatinine ratio, RK/Ao=Right renal length to aortic luminal diameter, LK/Ao=Left renal length to aortic luminal diameter, uPIIINP/Cr=Urinary procollagen type III amino-terminal propeptide to creatinine ratio. Data are presented as mean±SD. Statistically difference between groups was made using Unpaired t-test, **p<0.01; ***p<0.001. Correlations between parameters were made using Spearman correlation, *p<0.05; ***p<0.001

**Figure-2:**
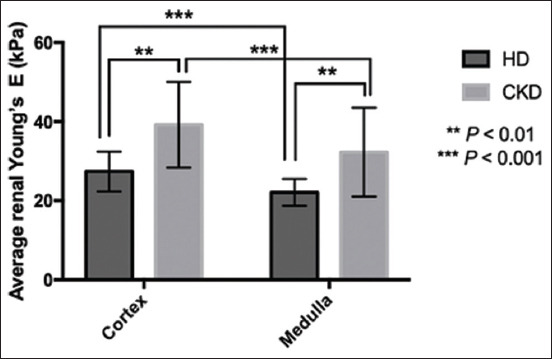
The average renal E values of cortex and medulla (mean±SD) within group and between groups of dogs; the healthy dogs and chronic kidney disease group. Statistical difference between the average renal E values of cortex and medullar within each group using Paired t-test; comparison between the average renal E values of cortex between groups using Unpaired t-test; comparison between the average renal E values of medulla between groups using Mann–Whitney test. ***p<0.001; **p<0.01.

The renal elasticity levels observed through SWE of both groups were reported as average E values calculated from either the cortex or medulla of the right and left kidneys (RK and LK). Both the average renal cortical and medullary E values of the right and left kidneys were not significantly difference in each group (Table-3). Both HD and CKD groups showed significantly higher of the average renal cortical E values than those of the renal medulla. In addition, when compared between groups, the average E values of both renal cortex and medulla in CKD dog were significantly higher than those of HD (Figure-2). Age, bodyweight, and body condition score (BCS) did not influence the average renal cortical and medullary E values of HD in this study. In addition, the average renal E values of cortex and medulla did not significantly differ between male and female dogs in the HD group.

**Table-3:** The average Young’s modulus (E) values (kPa) of renal parenchyma between HD and chronic CKD groups.

Group	E value of LK		E value of RK	
	
	Cortex	Medulla	Cortex	Medulla
HD	27.90±4.45	23.80±4.20	26.81±6.95	20.40±5.14
CKD	44.30±12.80	35.10±10.40	32.80±15.41	30.25±11.25

Data are expressed as mean±SD. The average E values between left and right kidneys in each group of dog were analyzed by Paired t-test. HD=Healthy dogs, CKD=Kidney disease dogs

The correlations between average renal E values and the functional renal parameters of all dogs are summarized in Table-4. The E values of both the cortex and medulla showed significant positive correlation with the concentrations of plasma Cr (p<0.001), BUN (cortex: p<0.01; medulla; p<0.05, respectively), UPC ratio (p<0.01), FE_Na_ (p<0.05), FE_K_ (p<0.05), FE_Cl_ (p<0.05), and FE_Mg_ (p<0.001) while they showed a negative correlation with USG (p<0.05). However, only the renal cortical E value presented a negative correlation with Uosm/Posm (p<0.05).

**Table-4:** The correlations between the average renal Young’s modulus (E) values and plasma Cr concentration, BUN, USG, UPC ratio, Uosm/Posm ratio and FE_Na_, FE_K_, FE_Cl_, FE_Mg_ in clinical HD and CKD dogs.

Parameter	Average E values of cortex	Average E values of medulla
	
	r	r
Plasma Cr	0.59***	0.62***
BUN	0.55**	0.43*
USG	−0.39*	−0.36*
UPC ratio	0.52**	0.51**
Uosm/Posm ratio	−0.41*	−0.32
FE_Na_	0.41*	0.44*
FE_K_	0.40*	0.39*
FE_Cl_	0.42*	0.41*
FE_Mg_	0.66***	0.58***

E value=The Young’s modulus values in kilopascal unit, BUN=Blood urea nitrogen, Cr=Creatinine, USG=Urine specific gravity, UPC ratio=Urine per creatinine ratio, Uosm/Posm ratio=Urine osmolality per plasma osmolality ratio, FE_Na_=Fractional excretion of sodium, FE_K_=Fractional excretion of potassium, FE_Cl_=Fractional excretion of chloride, FE_Mg_=Fractional excretion of magnesium, HD=Healthy dogs, CKD=Chronic kidney disease.

* p<0.05;

** p<0.01;

*** p<0.001

**Figure-3:**
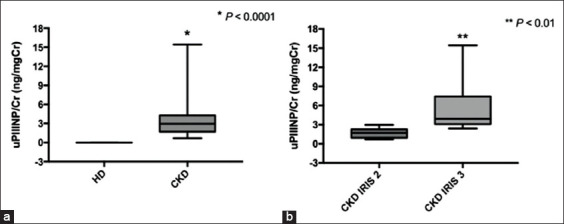
Box and whisker plot illustrating the uPIIINP/Cr ratios between groups using Mann–Whitney test; (a) the uPIIINP/Cr ratios between the HD and chronic kidney disease (CKD) dogs; (b) the uPIIINP/Cr ratios between CKD dogs with IRIS Stages 2 and 3. uPIIINP/Cr=Urinary procollagen type III amino-terminal propeptide to creatinine ratio, IRIS=The International Renal Interest Society staging system.

**Figure-4:**
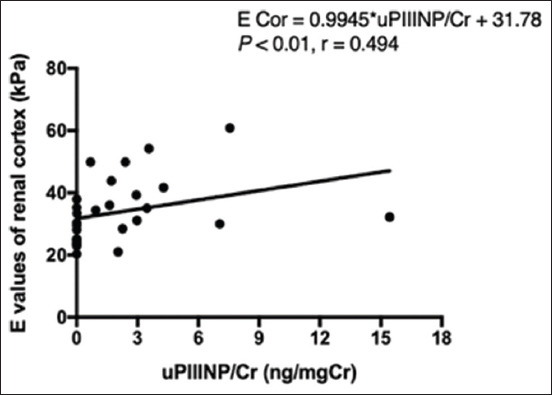
Correlation between average renal cortical E values from shear-wave elastography and uPIIINP/Cr of 30 dogs. Correlation was made using Spearman correlation coefficient. E=Young’s modulus values, uPIIINP/Cr=Urine Procollagen Type III N-Terminal Propeptide to creatinine.

For the uPIIINP/Cr ratios, the results showed that the average uPIIINP/Cr concentrations of CKD dogs were significantly higher than those of HD (mean uPIIINP/Cr of 3.930±3.740 and 0.004±0.003 ng/mgCr for CKD and HD dogs, respectively; p<0.001) (Figure-3a). The average uPIIINP/Cr ratio of CKD dogs with IRIS Stage 3 was significantly higher than that of CKD dogs with IRIS Stage 2 (mean uPIIINP/Cr of 4.468±2.026 and 1.752±0.780 ng/mgCr for IRIS Stage 3 and IRIS Stage 2 of dogs, respectively; p<0.01) (Figure-3b). A significant positive correlation (p<0.01) was found between uPIIINP/Cr ratios and average renal cortical E values (Figure-4), while uPIIINP/Cr ratios were not significantly correlated with average renal medullary E values. Moreover, the uPIIINP/Cr ratios showed positive correlations with the concentrations of plasma Cr (p<0.001), BUN (p<0.001), FE_Na_ (p<0.001), FE_K_ (p<0.001), FE_Cl_ (p<0.001), and FE_Mg_ (p<0.001), and the negative correlation with USG (p<0.001) and Uosm/Posm (p<0.001) (Table-2). No significant correlation was found between the uPIIINP/Cr and either the UPC ratio or blood pressure. In addition, the uPIIINP/Cr concentrations had significant negative correlations with both RK/Ao (p<0.05) and LK/Ao (p<0.05) (Table-2).

## Discussion

As knowledge is updated, it has been confirmed that the principle pathological alteration underlying the CKD advancement is renal fibrosis including glomerular and tubulointerstitial fibrosis. These impairments can eventually drive the progression of kidney diseases into end-stage renal disease (ESRD) [16,47]. Importantly, tubulointerstitial fibrosis is the strongest indicator of CKD progression [16,17]. At present, renal biopsy is the gold standard method for achieving a final diagnosis of renal fibrosis [16,28,48]. However, renal biopsy is an invasive procedure with several complications, most importantly, renal hemorrhage [49]. Therefore, the novel and non-invasive techniques or biomarkers that can represent morphological tissue alterations at early stages and can indicate an association with renal function impairment would be meaningful in predicting renal functional loss and disease progression in CKD dogs.

Ultrasonographic elastography is a diagnostic imaging modality that has been applied in human medicine for a few decades. This technique can differentiate pathological tissue from normal tissue by detecting the tissue elasticity; elasticity normally decreases through pathological alterations and becomes stiff especially due to fibrotic processes such as renal fibrosis in CKD patients [50]. Ultrasonographic elastography has been employed to assist in early CKD diagnoses and in detecting renal fibrosis progression of humans [36,37,50,51]. In veterinary medicine, studies involving this technique have been continuously increasing; several studies have been published concerning the evaluation of normal kidneys in HD [43,52] and cats [53], canine mammary tumor predictions [54,55], and comparisons between benign and malignant lymph nodes in dogs and cats [56] However, the information about the renal elastography has been reported only in cats with CKD [32]. It has not been any previously reported in CKD dogs.

SWE is the latest ultrasonographic elastography technique and it has several advantages over another technique called as strain elastography (SE). For example, SWE offers real-time monitoring of both structural and tissue stiffness information through a color quantitative elastogram overlaid on a B-mode image [57,58]. Moreover, the reproducibility of SWE between observers is also excellent [59]. Therefore, ultrasonographic elastography can effectively be used for non-invasive renal fibrosis detection and monitoring. Its use might reduce the need for renal biopsy procedures in humans [59]. As previous studies were conducted in humans, the finding in this study using SWE to investigate canine renal parenchyma elasticity in CKD dogs might assist the practitioners in the diagnosis and management of canine CKD.

In the present study, the renal length results from the B-mode ultrasonography showed that the renal length of CKD did not significantly differ from those of the HD. However, when compared the K/Ao ratio as described in the previous studied [24], both RK/Ao and LK/Ao ratios of CKD dogs were significantly lower than those of HD. The indifferent of average renal length in between groups is likely because our study included dogs of various breeds of dogs, leading to high variability in body conformation, and renal length. Mareschal *et al*. [24] were reported that using K/Ao is a suitable method for considering renal size in dogs. Our results corresponded with the previous studies in both dogs and cats [32,51,52], all of which suggest decreased that renal size is an important indicator in evaluation of CKD progression [24,51-53].

In agreement with the previous SWE study in cats [32], the renal E values between the right and left kidneys were not significantly different. Moreover, in both groups, our results showed that the renal cortex had the elasticity significantly lower than renal medulla which corresponds to the recent studies of using SWE to evaluate the renal elasticity in cat [32], pig [60], and also humans [37,40]. However, these results were different from the previous studied of using SE for evaluating kidneys in normal dogs [53] and cats [53]. These incongruous findings might be due to different elastography techniques being used among studies. Further comparative studies between elastography techniques should be conducted to provide more clinical data in evaluating the renal elasticity in dogs.

The renal elasticity of both the cortex and medulla in the CKD group was significantly lower than those of HD. Our findings correspond with the previous studies in both CKD cats [32] and humans [37,41,61,62] in that animals and people with CKD had stiffer kidneys than those of their healthy counterparts. However, our results showed an overlapping between the average renal E values of HD dogs and CKD dogs; this might currently limit SWE use as the definitive diagnosis tool for CKD. Accordingly, SWE should be used as a supplementary tool to acquire more accurate information for detecting and monitoring of canine CKD. Furthermore, SWE may assist practitioners in diagnosing and planning the proper treatment of renal disease in subclinical cases.

Age, gender, breed, bodyweight, and BCS had no significant effect on the renal E values of either cortex or medulla in this study, consistent with the previous findings in canine [43], feline [32], and human [63]. However, the small number of animals included in this study is one of the important limitations in evaluating the influence of demographic variables on SWE.

It is commonly accepted that renal tubulointerstitial fibrosis is the main final pathway of kidney disease [47] that is highly correlated with renal functional deterioration in humans [64,65], dogs [66], and cats [66,67]. In the present study, renal tubular functions were evaluated through the Uosm/Posm ratio, FE_Na_, FE_K_, FE_Cl_, and FE_Mg_ as a reflection of renal absorption and excretion of water and electrolytes. CKD dogs in this study presented aberrant urinary concentrating ability, contributing to the lower urine osmolality and Uosm/Posm ratio than those in HD. These results correspond with the findings of a previous study in cats [32], in which cats with CKD had lower urine osmolality and Uosm/Posm ratio than healthy cats. In addition, it has been reported that FE_e_ is a highly sensitive parameter for the assessment of renal tubular impairment in dogs with CKD [45]. Moreover, a study in humans revealed that FE_Mg_ showed a positive correlation with the degree of tubulointerstitial fibrosis; thus, it may be a useful marker for identifying the severity of renal tubular cell damage, especially of the renal proximal tubule [68].

For the relationships between the renal parenchyma elasticity and renal function parameters, the results demonstrate that the renal cortical E values or renal cortical elasticity was more significantly correlated with the renal functions than the renal E values of the medulla. In renal cortical elasticity correlations, the significantly positive correlations were observed with concentrations of plasma Cr, BUN, the UPC ratio, FE_Na_, FE_K_, FE_Cl_, and FE_Mg_. Meanwhile, the significantly negative correlations were observed between the renal cortical elasticity and either USG or the Uosm/Posm ratio. The results correspond with the prior studies in human [36] and also in cats [32] that decreasing renal elasticity in CKD patients was associated with progressive deteriorations of renal functions. These findings suggest that the renal cortical E value is more highly correlated with renal function compared with the renal medullary E value; this is consistent with a previous study in cats [32]. This may be because the renal cortex contains both the glomeruli, which are responsible for plasma filtration [69], and the proximal tubules, which are responsible for the reabsorption and secretion of substances, particularly of water and electrolytes [70].

Although urine osmolality and FE_e_ can represent the absorption and excretion capability of water and electrolytes, the results can be altered by many exogenous and endogenous factors, particularly diet, and hydration status [70]. To decrease these confounding effects in this study, we strictly evaluated the hydration status of all dogs by taking a complete history and performing physical examinations; only dogs with normal hydration were included in this study. However, the influence of food on these parameters could not be controlled in this study, because the use of a prescription diet is the clinical guidelines for CKD management. Therefore, this might be an additional limitation of this study.

The present study investigated the correlations of uPIIINP/Cr with the renal E values and the renal functional parameters due to the previous evidence indicating that uPIIINP/Cr ratio correlated with the severity of renal functional impairment and the degree of renal fibrosis in humans [16,29-31]. Moreover, uPIIINP levels also correlated with urinary levels of transforming growth factor beta 1 (TGF-β1) [30], which is commonly used as biomarker for renal fibrosis, assessment in humans and cats [71]. However, urinary TGF-β1 had no significant association with renal function impairment in either humans [48] or cats [72]. In humans, elevated uPIIINP levels could signify deteriorating renal tubular reabsorption ability due to renal fibrosis in CKD patients [16,29,30]. Moreover, a recent study showed that the uPIIINP/Cr ratio had a significant positive correlation with decreased renal parenchymal elasticity as detected by SWE in cats with CKD [32].

The uPIIINP/Cr ratios were significantly higher in CKD dogs than in HD, in agreement with the previous studies of CKD in humans [16,29,30], cats [32], and dogs with cardiac remodeling [33], idiopathic pulmonary fibrosis [34], and liver fibrosis [35]. In addition, the results showed no effects regarding the degree of proteinuria on the uPIIINP level in canine CKD similar to the previous studies in humans [30] and cats [32]. This maintains that there is no significant correlation between the uPIIINP/Cr ratio and UPC in those species.

Moreover, we also found that the average uPIIINP/Cr ratios were significantly higher in CKD dogs with IRIS Stage 3 than those in CKD dogs with IRIS Stage 2, with little overlap of the average uPIIINP/Cr ratios. This indicates that the uPIIINP/Cr ratio might be a good biomarker to use as an adjunctive tool for detecting the progression of CKD in dogs. Nevertheless, we did not include all CKD IRIS stages in this study, as it was difficult to categorize dogs with Stage 1 CKD by Cr only, and there were few Stage 4 CKD dogs; also some were not in a stable condition due to their poor physical health, especially if they had progressed to ESRD. Thus, recruiting dogs with all IRIS stages are challenging and may require the use of more laboratory parameters, such as serum or plasma symmetric dimethylarginine (SDMA) to differentiate IRIS Stages 1 and 2 [44].

In agreement with a previous feline study [32], a statistically positive correlation was found between the uPIIINP/Cr ratio and the average renal cortical E values which suggest that the renal cortical E value might be a promising indicator to assist in the evaluation of renal fibrosis in canine CKD.

In addition, the uPIIINP/Cr ratios had significantly positive correlations with the concentrations of plasma Cr, BUN, FE_Na_, FE_K_, FE_Cl_, and FE_Mg_, but showed significantly negative correlations with USG and Uosm/Posm. These results demonstrated that high uPIIINP/Cr ratios were associated with both glomerular and tubular renal functional deterioration. Our findings correspond with those of a study in humans with CKD reporting that uPIIINP correlated with blood Cr levels and the estimated GFR [16]. Moreover, uPIIINP/Cr correlated strongly with FE_Na_, FE_Cl_, and FE_Mg_, all of which are associated with proximal renal tubular function, and also had strong correlations with USG and Uosm/Posm, which are associated with a kidney’s ability to concentrate the urine.

At present, in veterinary medicine, information concerning renal function parameters and renal elasticity in dogs have not yet been reported. Moreover, the correlations between the uPIIINP/Cr ratio and renal elasticity in canine CKD have not been evaluated. This study is the first report of renal elasticity observed by SWE and the uPIIINP/Cr in CKD dogs. This information would be useful for clinical practitioners and may be beneficial for further studies.

Aside from the aforementioned dietary limitations, a main limitation of this study is that renal histopathology was not investigated in the comparison of renal elasticity determined by SWE and uPIIINP/Cr ratios due to the impracticality and invasiveness of renal biopsies. Furthermore, the small number of included dogs in the study was not adequate to determine the influence of demographic variables on SWE.

## Conclusion

SWE can be utilized as an additional diagnostic imaging tool for evaluating renal elasticity in CKD dogs since elasticity is significantly correlated with renal functional parameters. The renal cortex had lower elasticity than the renal medulla in both HD and CKD groups; also, the kidneys of dogs in the CKD group were stiffer than those of HD in this study. In addition, the renal cortical E values were significantly correlated with the uPIIINP/Cr ratios. Furthermore, the uPIIINP/Cr ratios were higher in CKD dogs. Therefore, the uPIIINP/Cr might also be a promising biomarker for a complementary evaluation of renal fibrosis in canine CKD. However, further studies are needed to evaluate the clinical E value of SWE for evaluating and monitoring dogs with CKD and to explore the relationship between uPIIINP/Cr ratios and different stages of renal fibrosis in dogs. In the future, SWE and uPIIINP/Cr ratios might be useful in animals, in which a renal biopsy is contraindicated.

## Authors’ Contributions

Study conception and design: CT, SS, CB, and NC; acquisition of data: CT and NC; laboratory test: CT and CB; analysis and interpretation of data: CT, SS, CB, and NC; drafting of manuscript: CT, SS and NC; critical revision: CT, SS, CB, and NC; all authors read and approved the final manuscript.
